# Practical Observations on Aural Surgery and the Nature and Treatment of Diseases of the Ear

**Published:** 1855-02

**Authors:** 


Art. II.—*Practical Observations on Aural Surgery and the Nature and Treatment of Diseases of the Ear.* With Illustrations. By William R. Wilde; Fellow of the Royal College of Surgeons in Ireland; Surgeon to St. Mark's Ophthalmic Hospital. Philadelphia: Blanchard & Lea. 1853. pp. 475, 8vo. [*Continued from the January number.*]

The condition of the digestive organs exerts less influence over inflammatory affections of the ear than those of the eye. The bowels, however, in this as in all other febrile diseases should be kept open. The use of tne means calculated to allay inflammation and febrile disturbance, should be had recourse to. The skin being generally hot and dry, sudorifics, are, in the early stage of the disease, decidedly indicated. Having leeched, fomented and purged, James's powder, combined with small doses of blue pill and henbane, will be found very efficacious.

In the more advanced stage of the disease and after proper depletion, blisters are highly advantageous.

The symptoms remaining unrelieved after the employment of all these means, mercury should at once be had recourse to. Our author is so fully convinced not only of the utility, but of the urgent necessity of employing mercury in these aural inflammations, that he does not hesitate to recommend its use in the early stages of all such affections. When it is employed early, it produces, as soon as its constitutional effects are manifest, a well marked improvement in all the symptoms. The patient should be kept under its gentle influence for some days.

The speculum should be used daily. If an ulcer be discovered it should be touched with a solution of nitrate of silver. If otorrhea occur, the discharge should be removed by very gently syringing the part with simple warm water. No stringent injections whatever should be employed while the inflammatory action runs high.

*Subacute myringitis* though a perfectly painless disease, is equally destructive to hearing. Persons between fifteen and thirty are most subject to it. Deafness, coming on rather suddenly, is often the first symptom noticed by the patient. The auditory canal does not usually exhibit signs of disease, but the ceruminous secretion is arrested. When seen early, the membrane is always of a pink color, of a tint somewhat paler than that of the monthly rose; its transparency and polish are seldom much affected at first. If treatment be not resorted to, the membrane becomes thickened, and opaque, from deposits of lymph, and irremediable deafness ensues. Mercury is as essential in this disease as in acute myringitis. To be effectual however, it must be slowly introduced into the system, so as to produce a steady and gradual effect, which should be kept up "until there is a decided improvement both in the vascularity and hearing or until all hope of restoration has been abandoned. After the constitution has been fully affected by the mineral, the bichloride, given in doses from the sixteenth to the eighth of a grain, dissolved in proof spirits, and taken in half an ounce of the cold infusion of bark, a scruple or half a drachm of Huxham's tincture, three times a day, will be found highly efficacious. The preparations of iodine are also, in the advanced stages of the disease, worthy of trial; but I do not think that the preparations and combinations of iron produce in aural inflammations the same benefit which they do in constitutions laboring under ophthalmic affections of a like character."

"To relieve tinnitus aurium, after the inflammatory action has been subdued, or the original disease which produced it has subsided, and particularly in cases where we find this symptom present without any apparent lesion of the parts we are able to inspect, I have found the preparations of the Arnica montana of decided benefit; indeed it is the only medicine with which I am acquainted that seems to possess a specific power over this annoying and usually most intractable complaint. The preparation I find most efficacious is the tincture both of the flowers and leaves, of which the patient should commence by taking fifteen drops in a tablespoonful of the infusion of Arnica, with some cordial tincture three times a day. After a few days the dose should be increased one or two drops daily, till it reaches thirty, or even more, unless headache or giddiness be produced, when we should at once lessen the dose, or omit the medicine altogether for a short time. The state of the bowels should be carefully attended to during the administration of this drug."

*Syphilitic myringitis*, *gouty otitis*, *strumous myringitis*, *otitis in connection with ophthalmia, and typhoid and exanthematous inflammations of the membrana tympani*, are next treated of in a most lucid and satisfactory manner.

*Chronic myringitis* is a very frequent cause of deafness. In the tables of our author no less than one in every six cases of deafness were attributable to this cause.

There are two special forms of chronic myringitis that constantly present themselves—one a perfectly painless deafness; the other attended by paroxysms of pain, occurring at intervals, between which the patient is entirely free from all uneasiness. "The latter is much more common among females from twenty to forty, and is at times accompanied by irregularities of the uterine functions. The appearance of the membrana tympani is too peculiar to be mistaken; it presents a general thickening and opacity, particularly of its lower portion; besided which there is almost invariably a number of spots, about the size of pin heads, of greater density than the rest, and of a pearly lustre, studded over the surface of the membrane. In many cases it presents the appearance of crumpled parchment. During the quiescent periods, we only remark a few straggling vessels, carrying red blood, spread over the surface of the membrane, and, for the most part coursing from above downwards, parallel with the handle of the hammer. Upon any provocation, however, such as cold, or other exciting causes, the membrane will, in a few hours, and often without any increase of pain, become an uniform dark-red color, precisely like *pannus* of the cornea, a disease of which it is the manifest analogue. The gr ater the amount of thickening and opacity, the less will be the quantity of vascularity and redness which the membrane is capable of assuming, as we perceive in cases of dense opacity of the cornea, owing, no doubt, to the greater quantity of deposit obstructing the flow of red blood, by diminishing, and, perhaps, also obliterating, the calibre of the vessels. In such cases the membrane is often insensible."

Little can be done in such a case. Lunar caustic, in solution, applied every third or fourth day, upon the opaque membrane, and if there be much vascularity present, the application of a few leeches, twice or thrice a week, constitute the only means that are likely to be serviceable.

"In the cases of periodic pain, with a higher degree and more generally diffused vascularity, the application of leeches, every second or third day, will be found most efficacious, at the same time that the patient should be brought under the gentle influence of mercury, and kept so for at least a month."

The most frequent and apparent result of each and all of the foregoing varieties of inflammation in that structure, says Mr. WILDE, is thickening and opacity of the membrana tympani. This condition of the membrane is as difficult of cure as the same appearance in the cornea. Counter irritation over the mastoid process, and painting the surface of the membrane with a solution of lunar caustic, of from ten to twenty grains to the ounce, twice a week compose the staple of the treatment. Our author has tried iodine and other substances, but gives nitrate of silver the preference.

Artificial preforation of the membrana tympani is not regarded by Mr. WILDE as being of either such general use or applicability as is commonly believed. He remarks of it, "there has not been, perhaps, in the whole history of medicine during the present century a discovery to which so much praise was at the time awarded, that subsequent investigation and experience have, to say the least of of it, so much disparaged."

The operation was performed by ITARD in a great number of instances, but without any determined beneficial result. The only true indication for the preforation of the membrana tympani, is, according to KRAMER, afforded by the thickening of the membrane, unaccompanied by any other disease of the ear. Mr. WILDE thinks there are very few cases, indeed, in which it will be found efficacious. The operation requires very great caution and dexterity in its performance. When it becomes necessary our author suggests the following as the best method to be adopted.

The membrane is first to be brought fairly in view, under bright, direct sunlight, and the patient made to inflate the tympanum, so as to render the membrane tense. The point of a small sickle-shaped knife, with a double cutting edge, is then introduced into the inferior, thin, vibrating portion, and drawn downwards and forwards, and a simple incision of the membrane, about a line and a half in length, accomplished. This procedure is so simple and painless, that the patient is often unconscious of its performance until made aware of its completion by the air rushing out through the aperture. If left in this condition, the wound would soon heal up; therefore, a very fine probe, fixed in a handle, and slightly pointed with nitrate of silver by being immersed in the caustic when heated to fluidity, should be immediately passed down into the perforation, the edges of which are thereby cauterized and prevented adhering; and this latter process should be repeated from time to time, as often as the wound shows an inclination to heal, and until we establish a sufficiently large elliptical opening.

Accidental perforation of the tympanal membrane may occur from a variety of causes. When the result of inflammation, otitis is the form of disease which most frequently gives rise to it.

The cause, duration and extent of the aperture will in a great degree, determine the success of the treatment—if produced by ulceration it is more unpromising than when the result of mechanical injury, or accumulated fluid.

Our author after a long and fair trial of several means proposed for the cure of these perforations, believes there is nothing like nitrate of silver. In order to derive the full benefit of the escharotic, it should be applied by means of a probe, "upon the extreme edge of the aperture, or rather within the ring of the opening, every second or third day, so long as the part seems inclined to close, but the moment we perceive it enlarging, a day or two after the application, we must desist."

*Acute otitis* is an affection the causes and symptoms of which it is not necessary for us to describe in this place. They are familiar to every practitioner.

The terminations of the disease are not so well understood. They are three fold:—First, resolution; Second, rupture of the tympanal membrane; and Third, the inflammatory process spreads from the cavity of the tympanum to the mastoid process, producing disease of that bone. The last, it need not be remarked, is a matter of no little danger.

The treatment advised in acute myringitis, carried to the fullest extent, is imperatively demanded in acute otitis. Mr. Wilde regards mercury even more necessary here than in myringitis; it should be commenced at once and with a two-fold object; to arrest the disease in the ear, and should it fail in so doing, to check its inward progress to the brain.

*Exanthematous otitis* derives great interest from the affections with which it is associated. It occurs more frequently perhaps in scarlatina than any other of the exanthema, and our author states that the most unmanageable cases of otorrhœa with which he has ever met, those in which most destruction has taken place, and where the ossicula have been most frequently lost, have been the result of scarlatina or measles.

The remainder of chapter vii. is devoted to chronic otitis, facial paralysis, diseases of the Eustachian tube, throat deafness, &c. We have not space to do more than mention these affections.

*Nervous deafness* occupies the greater part of chapter VII. Our author regards this as a disease much more rarely encountered than is usually stated in the books. It generally occurs between twenty and thirty-five years of age—usually commences on one side, but sooner or later extends to both ears—patients invariably hear worse on being in any way excited—tinnitus is a frequent but not an invariable attendant, &c.

Nervous deafness must be treated according to its cause. Almost every mode of treatment has been advised. Kramer is in the habit of introducing the vapor of acetous ether into the tympanic cavity through the Eustachian cavity. Other aurists recommend purgation and acting on the mucous membrane; others, again, use emetics; others mercury, and others electricity and galvanism. Mr. Wilde declares however, that none of these various and manifold modes have had the desired effect.

For confirmed cases, our author has nothing to offer but an ear trumpet and a strong recommendation not to quack. For incipient nervous deafness, in which he thinks much may be done, if not to restore the hearing, at least to arrest further progress of the disease, and avert total deafness, he advises long continued counter irritation, the judicious use of mercury persevered in for months, and attention to the general health. With the exception of counter irritants behind the ears, Mr. Wilde entreats his readers carefully to abstain from all topical applications.

The concluding chapter of the work treats of *Otorrhea*, and we intended in the outset, to have given considerable space to this subject, but we have already extended our notice to perhaps greater length than is either judicious or desirable. We cannot refrain, however, from sketching the outlines of this most interesting disease, even at the risk of wearying the patience of some of our readers.

Mr. Wilde says otorrhœa, or a discharge from the ear, is the most frequent aural disease of Great Britain. The same, we believe, may be said of the affection in this country. In the tables, to which we have already referred, it amounted to 647 in 2385 cases registered from all causes, or about 1 in every 3½. It is a disease of infancy and youth; it seldom appears in middle life, and still less frequently in advanced age. The principal causes of otorrhea have been enumerated in our remarks on the various inflammatory affections of the ear. Our author believes if there is one disease more than another that shows a strumous constitutional taint, it is otorrhœa, particularly that form of it which appears in young persons and children without any apparent cause.

Otorrhœa may be produced during the progress of scarlatina and measles—either by direct extension of the inflammation of the skin into the external meatus and tympanal membrane; by the diseased condition of the mucous membrane of the throat passing up through the Eustachian tube, or, finally, by the abscesses which occur in the neck and around the meatus, opening into the fibro-cartilaginous portion of that tube.

Two questions of vital importance in the treatment of otorrhœa present themselves:—one as to *prognosis;* the other, as to the *morbid changes* to which long neglected aural discharges may lead. The prognosis should always be cautious, unless we see our way through the case very clearly, for the reason that, "*so long as otorrhœa is present, we can never tell how, when, or where*

*it will end, or what it may lead to.*" Mr. WILDE remarks whether the disease has arisen from constitutional taint, as a painless running on the one hand; or whether it be the result of scarlatina, owing to acute inflammation, and suppuration of the tympanum, on the other; it is always difficult to manage. If the case be of long standing, the discharge becomes like that which arises from a fistula, and its treatment is very tedious; and in no case should the patient be promised permanent relief until we have assured ourselves of the total extent of the parts engaged.

The *treatment* in simple external otorrhœa as found most useful by our author, consists in paining the surface engage with a solution of nitrate of silver, ten grains to the ounce, with a fine camel's hair pencil, or, preferably a bit of cotton on the end of a probe. This should be repeated every second day. Syringing the ear with plain tepid water night and morning for the purpose of ensuring cleanliness, should be practised; "and at night a slightly astringent lotion may be poured into the ear until it fills up the meatus, and allowed to remain there for a few minutes with the head bent to the opposite side, and then permitted to run out. The syringing, must not, however, be overdone; as soon as the discharge begins to moderate, it should be had recourse to less frequently."

"The various salts which enter into the general composition of eye collyria are here particularly applicable, especially those of alum, lead, zinc, and copper. I formerly applied the lead lotion very extensively, but I have found that it frequently, even without coming in contact with diseased bone, produces a blackened discharge: when it is used, the liquor plumbi will be found the safest and most efficacious preparation; and the lotion may be preserved clear, and either rose or elder-flower water employed, by the addition of a few drops of acetic acid. The liquor aluminis composition of the London Pharmacopœia is that which I now most frequently prescribe. Solutions of tannin will also be found useful astringents.

If upon examination we find the meatus thickened, and it and the surface of the membrana tympani pink and vascular, a leech or two, according to the age and strength of the patient, should be applied every third day, several times. When the discharge is fetid, a chloride of lime lotion used occasionally is of service, being slightly astringent, and correcting the disagreable smell."

In regard to the constitutional treatment, if we have reason to believe, that the state of the general health assists to keep up the local disease, our author recommends cod-liver oil and Peruvian bark: the former as an anti-strumous fattener; the latter by altering the established tendency to morbid secretion. Mr. Wilde has seldom found it necessary, however, to prescribe any course of alterative medicine, unless in cases of marked strumous habit, and when the glands of the neck are diseased. If the aural discharge has broke out or the subsidence of disease of the skin or any vicarious outlet, or the child has had convulsions in infancy, or disease of the brain has appeared in other members of the family and their like exceptional conditions, "drains" and counter irritation may be required. Under ordinary circumstances our author does not advise them.

Small abscesses sometimes form round the mouth of the meatus and fresh attacks of otalgia occur towards the close of an otorrhœa from simple chronic otitis, especially in children. Mr. Wilde regards these as "best warded off by the application of a vesicating liniment behind the ears, and keeping up gentle counter-irritation for some little time after the otorrhœa has ceased. For this purpose the croton oil dissolved in soap liniment, or the tincture of iodine, made stronger and more soluble by the addition of a little hydriodate of potash or the acetum lyttœ, answers very well."

In cases of long standing otorrhœa, when the discharge is ceasing, either spontaneously, or as the result of treatment, in very many instances the dermal lining will be found to have become enormously thickened; and the cuticle to be thrown off in patches, sometimes filling up the passage completely. This condition of things will in most instances be quickly righted by first syringing the ear well and removing all the thickened cuticle that remains through the speculum, with the spatula and a pair of slender forceps; "the parts may then be touched with a solution of lunar caustic, and when the discharge has ceased, and the cuticle has become thinner and less white, the cure may be completed by the application of ung. hydrarg. nitros dil., laid on warm with a camel's hair brush."

Morbid vascular growths are among the complications that render otorrhœa at all times tedious and difficult to heal. Of these, *Granulations covering over the face of the membrana*

*tympani* are set down by our author as not being unfrequent. The solid nitrate of silver rubbed over them every second day, or oftener, if necessary, is esteemed by Mr. WILDE as the best means for their removal.

Polypus, caries and cerebral affections consequent upon otorrhœa engross the remainder of the chapter. D. W. Y.

